# *Notes from the Field:* Multistate Outbreak of *Salmonella* Agbeni Associated with Consumption of Raw Cake Mix — Five States, 2018

**DOI:** 10.15585/mmwr.mm6834a5

**Published:** 2019-08-30

**Authors:** Stephen G. Ladd-Wilson, Karim Morey, Sarah E. Koske, Bailey Burkhalter, Lyndsay Bottichio, Joshua Brandenburg, John Fontana, Kristina Tenney, Kirthi K. Kutumbaka, Mansour Samadpour, Katherine Kreil, Paul R. Cieslak

**Affiliations:** ^1^Oregon Health Authority, Public Health Division; ^2^Oregon Health Authority, Oregon State Public Health Laboratory; ^3^Wisconsin Division of Public Health; ^4^Douglas County Public Health, Roseburg, Oregon; ^5^National Center for Emerging Zoonotic Infectious Diseases, CDC; ^6^Institute for Environmental Health Laboratories, Lake Forest Park, Washington; ^7^Coordinated Outbreak Response and Evaluation Network, Food and Drug Administration, Silver Spring, Maryland.

In August 2018, two Oregon patients with diagnosed *Salmonella* infection were interviewed using a standard enteric illness questionnaire; both patients reported having eaten raw cake mix. Standardized interview questionnaire data collected from 207 Oregon patients with salmonellosis in 2017 indicated a 5% rate of consumption of raw “cake mix or cornbread mix” (Oregon Health Authority, unpublished data, 2017). The binomial probability that both 2018 patients were exposed to raw cake mix by chance was determined to be 0.003, prompting the Oregon Health Authority (OHA) to collect and test the contents of 43 boxes of unopened cake mix of various brands from six retail locations. OHA sent samples to the Institute for Environmental Health Laboratories in Lake Forest Park, Washington, for pathogen testing. *Salmonella* Agbeni was isolated from an unopened box of white cake mix from manufacturer A, and whole genome sequencing (WGS) data describing the isolate were uploaded to the U.S. National Library of Medicine’s National Center for Biotechnology Information (NCBI) website (https://www.ncbi.nlm.nih.gov/pathogens). OHA used the NCBI database to compare sequence data with the cake mix isolate (PNUSAS056022) and then consulted CDC’s System for Enteric Disease Response, Investigation, and Coordination (SEDRIC), a web-based, outbreak investigation tool designed for collaborative, multistate investigations of enteric disease outbreaks.* On October 19, OHA determined that clinical isolates from four patients from Maryland, Ohio, and Wisconsin, with specimen isolation dates ranging from June to September 2018, were genetically related to the *Salmonella* Agbeni isolate from the unopened box of white cake mix, within four single nucleotide polymorphisms (SNPs).

On October 22, 2018, OHA notified state public health counterparts in the three states of this finding and inquired about raw cake mix exposures among their patients. The Wisconsin patient reported having consumed an entire box of raw white cake mix over several days during the likely exposure period. In addition, WGS analysis indicated that this clinical isolate was closely related genetically (within one SNP) to the isolate cultured from the Oregon white cake mix. On October 25, CDC requested officials in Maryland, Ohio, and Wisconsin to interview patients using a questionnaire with specific questions about baking exposures.

On October 31, the Food and Drug Administration (FDA) initiated an investigation of manufacturer A with regard to the *Salmonella*-positive white cake mix. In addition to the investigation and document collection, FDA collected samples including an ingredient (flour), finished cake mix, and environmental samples. All collected samples tested negative for *Salmonella.* On November 5, a voluntary recall of manufacturer A’s classic white, classic butter golden, signature confetti, and classic yellow cake mixes was announced because they might be contaminated with *Salmonella* bacteria.

On January 14, 2019, CDC declared this outbreak, which totaled seven cases in five states,^†^ to be over (*1*). This is the first time that OHA used WGS data on the publicly available NCBI website to detect a multistate outbreak associated with a widely distributed consumer product, which resulted in product action. WGS of food and environmental isolates and subsequent analysis on the NCBI and SEDRIC platforms are emerging as useful tools in identifying outbreaks associated with widely distributed products with long shelf lives and low background rates of consumption, such as raw cake mix. Detection of these outbreaks is typically difficult and relies mainly upon epidemiologic evidence from investigation of a larger number of cases (*2*–*4*). These efforts also highlight the value of collaboration between public health epidemiologists and laboratorians as well as the use of new technological tools for outbreak detection. During outbreak or cluster investigations, food and environmental samples should be collected as quickly as possible whenever practical, particularly when epidemiologic data suggest an association. WGS, in conjunction with the NCBI website and SEDRIC, can be used to identify genetically related isolates quickly.

## Footnotes

* https://www.cdc.gov/foodsafety/outbreaks/investigating-outbreaks/sedric.html.

† Florida, Maryland, Missouri, Ohio, and Wisconsin.
